# Physicochemical Properties of Dried Apple Slices: Impact of Osmo-Dehydration, Sonication, and Drying Methods

**DOI:** 10.3390/molecules25051078

**Published:** 2020-02-28

**Authors:** Joanna Cichowska-Bogusz, Adam Figiel, Angel Antonio Carbonell-Barrachina, Marta Pasławska, Dorota Witrowa-Rajchert

**Affiliations:** 1 Department of Food Engineering and Process Management, Warsaw University of Life Sciences, 02-776 Warsaw, Poland; dorota_witrowa_rajchert@sggw.pl; 2 Institute of Agricultural Engineering, Wrocław University of Environmental and Life Sciences, 51-630 Wroclaw, Poland; adam.figiel@upwr.edu.pl (A.F.); marta.paslawska@upwr.edu.pl (M.P.); 3 Agro-Food Technology Department, Food Quality and Safety Group, Miguel Hernández University, Carretera de Beniel, Orihuela, Alicante 03312, Spain; angel.carbonell@umh.es

**Keywords:** convective drying, vacuum-microwave drying, color, hygroscopic properties, sensory analysis, chemical analysis

## Abstract

Apple slices of the Elise variety were previously osmo-dehydrated in erythritol, xylitol, and sucrose for 2 h. In some parts of the experiment, 30 min of ultrasound pre-treatment (US) were applied. Afterwards, fruit samples were dried by convective (CD), microwave-vacuum (VM), and a combined method (CD/VM, mix two of them). The main aim of the research was to characterize an impact of osmotic dehydration, sonication pre-treatment, and drying method on the physicochemical properties of the dried apples. The use of sugar alcohols (xylitol, erythritol) in the production of dried apples did not badly affect the taste of the obtained dried products; it enabled a noticeable cooling/refreshing effect felt in the mouth when consuming a snack, and enabled the production of dried snacks with lower calorific value. Polyol residues in the product were at a level that was safe for consumers. The most popular convective drying was long lasting, whereas the VM drying method allowed for the shortest drying time, amounting to 76 min; moreover, additional application of ultrasounds reduced this time to 36 min. The combined drying method allowed the total duration of the process to be reduced 2–4.5 times. Ultrasound applied during osmotic dehydration did not significantly affect attributes of the descriptive sensory analysis for the obtained dried apples. The best hygroscopic properties, ensuring the storage stability of the dried product, showed dried apples previously osmo-dehydrated in erythritol and sucrose solutions.

## 1. Introduction

Apples are a natural source of sugars and dietary fiber (80% of which are soluble fibers), as well as various minerals, and vitamins. Consumers prefer dried apples to be as close as possible—in eating quality—to a fresh apple. Thus, the texture, color, and taste of dried apples are important [1]. Scientists and innovative food centers are looking for emerging food processing technologies to enable the introduction of new, safer, fresher, and better quality foods, with longer life for local and export markets. Among emerging technologies, ultrasonic dehydration is very promising, because the effects of power ultrasound are more significant at low temperature, which reduces the probability of food degradation [2]. Forced convection drying (CD) is one of the most common drying operations in the food industry and, together with ultrasound wave applied during the process, shortens the total drying time [3]. Vacuum-microwave (VM) drying of fruits is becoming more popular because of its advantages. In this method, microwaves penetrate the interior of the material subjected to drying, causing water to boil at low temperatures. This creates a large vapor pressure, differential between the center and the surface of the material, allowing rapid transport of moisture out of the product and preventing structural collapse [4]. A combined drying method (CD/VM), which includes convective pre-drying and microwave-vacuum finish-drying, shortens the drying time by about 50%, compared to other methods used for drying apple cubes [5]. Osmotic dehydration is the process that can be used as pre-treatment for conventional drying procedures. Removal of water and solutes in the food, as well as osmotic solute gain, are simultaneous isothermal flows without any phase change. The osmotic dehydration process provides the possibility of modifying properties of food with the aim of improving the quality of the final products [6]. Sugar alcohols could be an alternative to commercial sucrose osmotic agents in this process [7]. Moreover, additional sonication enhances efficiency (e.g., by reducing water activity) [8].

The aim of this study was to investigate the effect of osmotic dehydration, sonication pre-treatment, and the drying method on physicochemical properties of dried apples. To estimate that impact, drying kinetics were the model, and the following determinations were conducted: dry matter content with water activity, hygroscopicity, color changes, selected chemical analysis, as well as descriptive sensory analysis. The product safety of dried apple snacks (containing the rest of sugar alcohols, understood as not exceeding the dose resulting in gastric problems) was also estimated.

## 2. Results and Discussion

### 2.1. Drying Kinetics

**Figure 1:**
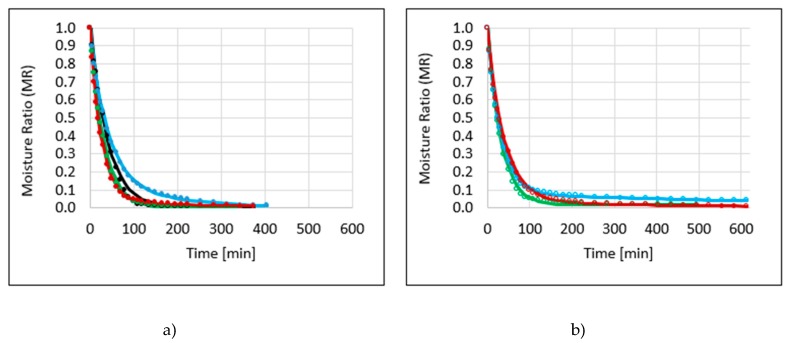
Drying kinetics of apple samples by the convective method (CD), previously osmo-dehydrated in erythritol (blue line), xylitol (green line), sucrose (red line), and without osmotic dehydration (OD) (black line), without application of ultrasound (**a**) or with ultrasound (US) pre-treatment (**b**). Parameters of drying models and statistical values of root-mean-square error (RMSE) and R^2^ are presented in Table 1.

The drying course is presented as MR (ratio of the actual moisture content to the initial moisture content—Equation (3)) changes at the time. Drying kinetics of apple samples osmo-dehydrated (or not) in different polyols solutions obtained by the convective method are shown in Figure 1a, b. Osmotic dehydration before drying had significant influence on the total drying time (Figure 1a, Table 1). The longest process of convective drying was in the case of erythritol, used as hypertonic solution (ECD)—405 min, and the shortest time was observed in the case of xylitol (XCD)—315 min, which allowed to shorten the total time of drying of half an hour, compared to control samples (RCD). Nomenclature of all of the samples is presented in Table A1.

The use of additional sonication during osmotic dehydration resulted in a significant extension of the duration of the convective drying process, from 2.5 h to 4 h, in the case of xylitol and sucrose solutions, respectively (Figure 1b, Table 1). Based on these observations, the impact of the use of ultrasound as pre-treatment was found to be unfavorable on convection drying after osmotic dehydration. The same observation was reported by Mierzwa and Kowalski [9], the ultrasound positively affects the drying kinetics for fresh—not pretreated—osmotic dehydration (OD) or ultrasound (US)-assisted OD samples. Unfortunately, the application of ultrasound did not influence the kinetics of initially osmo-dehydrated convectively dried samples. Researchers explain that this negative outcome was probably caused by sugar, which penetrated the material during osmotic processes. However, time reduction of convective drying time after application of the ultrasound was noticed in the literature for apple slices by 5–13% [10], and 27% for apple cubes [3] in relation to untreated material, but this material was not osmotic dehydrated before drying.

**Figure 2:**
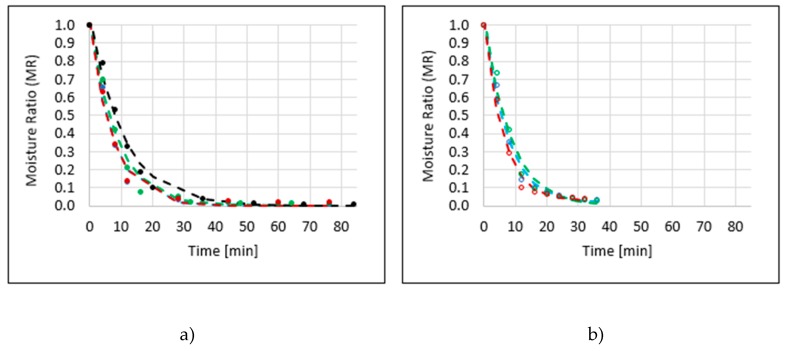
Drying kinetics of apple samples by the microwave-vacuum method (VM), previously osmo-dehydrated in erythritol (blue line), xylitol (green line), sucrose (red line), and without OD (black line), without application of ultrasound (**a**) or with US pre-treatment (**b**). Parameters of drying models and statistical values of RMSE and R^2^ are presented in Table 1.

Another technique for drying apples was the microwave-vacuum method, which enabled a significant reduction of the process time to 76 min in the case of osmo-dehydrated apples in three variants (erythritol, xylitol, sucrose) and to 84 min in the case of control sample (Table 1). The drying kinetics of EVM (all abbreviations—Table A1) apples almost coincided with the kinetics of dried SVM, while being similar to the kinetics obtained for dried XVM (Figure 2a). The use of additional sonication during osmotic dehydration had a beneficial effect on the course of microwave-vacuum drying. The process time was shortened to dehydration, 36 min in the case of apples, which were dehydrated in polyol solutions, and to 32 min in the case of a sucrose solution (Figure 2b), which was the shortest of the noted drying times (Table 1). Fuente-Blanco et al. [2] noticed an influence of ultrasound on the creation of microscopic channels, reducing the diffusion boundary layer, and increasing the convective mass transfer in foodstuff. Ultrasonic waves increased the effective water diffusivity because water could use these microscopic channels as an easier pathway to diffuse towards the surface of the fruit. On the other hand, the osmotic dehydration increased the effective water diffusivity by breaking down part of the cell walls, reducing the resistance for water to diffuse through the cells [11].

**Figure 3:**
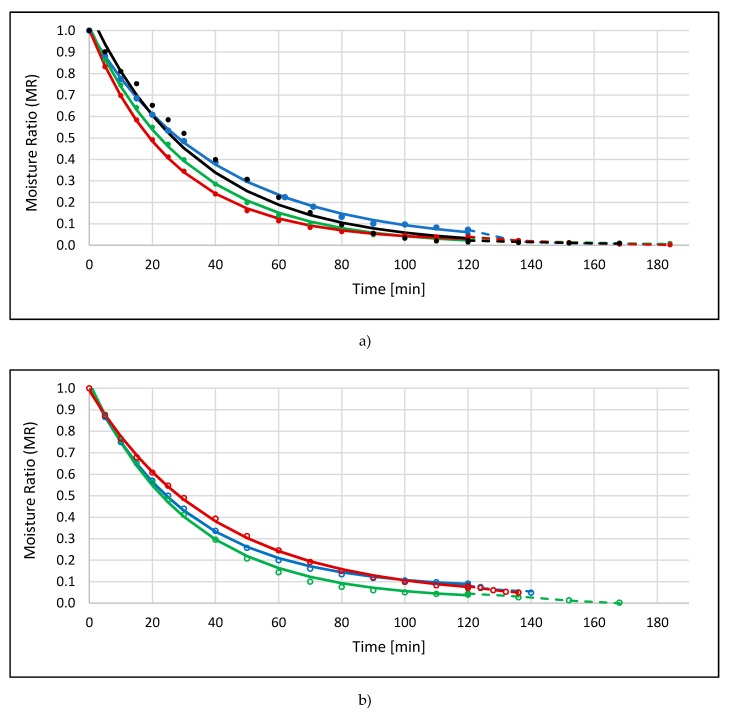
Drying kinetics of apple samples by the combined method (CD/VM), previously osmo-dehydrated in erythritol (blue line), xylitol (green line), sucrose (red line), and without OD (black line), without application of ultrasound (**a**) or with US pre-treatment (**b**). Solid lines are model of convective pre-drying; dashed lines are model of microwave-vacuum finish drying. Parameters of drying models and statistical values of RMSE and R^2^ are presented in Table 1.

The last used technique was combined drying (CD/VM), consisting of convective drying and microwave-vacuum drying. After elaborating the results, it was found that convective pre-drying took too long; as a result, the water content in the dried apples before VM drying was very low, and the reduction of the entire process time did not look as spectacular as in the case of tests carried out by Figiel [12]. The main difficulty of the CD/VM method concerns the times when CD should be replaced by VM drying. As the main role of convective pre-drying lies on maximal water removal (from the raw material) with a satisfactory drying rate (which represents the rate of MR change in the time unit), VM should start at a critical point of CD when drying kinetics is losing its linear character for the exponential decrease of moisture content in time characterized by the decreasing drying rate [13]. Presumably, in the meantime, one of the magnetrons was also turned off while the dryer was working at “half power”; it is not known when this situation began to affect the results. Figure 3 shows the kinetics of the combined drying of apples dehydrated in different solutions without (Figure 3a) and using ultrasound (Figure 3b). Comparing the total drying time (Table 1) of apple samples previously subjected to erythritol (blue lines), it can be stated that the combination of convective and microwave-vacuum drying reduced the drying time more than twice in the non-sonication option (from 405 to 184 min) and more than four times in the case with ultrasound pre-treatment (from 615 to 140 min). This observation is in agreement with other research [14], where it was reported that finish-drying of the apples with the VM method preliminary dried with the convective method, as well as considerably shortened the total time of drying, and reduced drying shrinkage.

Similar kinetics of combined drying was noted in the case of apples dehydrated in xylitol solution—the total drying time without US was also 184 min (Figure 3a, Table 1). The additional application of ultrasound allowed for reducing the process time to 168 min (Figure 3b, Table 1), but it was smaller compared to the previously discussed example of ECD/VM_US. Microwave-vacuum finish drying of apples osmo-dehydrated in a sucrose solution (SCD/VM) also lasted 184 min, which proves that the type of osmotic substance did not affect the length of combined drying time in variants without sonication. Pre-treatment of ultrasound during OD enabled the largest reduction of drying time, even 4.5 times (from 615 to 136 min) (Figure 3b). The combined drying method, also in the case of the control sample (black line in Figure 3a), allowed reduction by twice the drying time, compared to the convection (Table 1).

According to experimental data obtained during drying by three methods (CD, VM, CD/VM), it was stated that the loss of water content in apple samples could be described by means of a two-term exponential model function (Equation (1)):

(1) $$MR=b_{1}\cdot e^{-k_{1}\cdot t}+b_{2}\cdot e^{-k_{2}\cdot t}.$$

or by an exponential function, Henderson–Pabis model (Equation (2)):

(2) $$MR=b_{1}\cdot e^{-k_{1}\cdot t}$$

These models were chosen based on previous research, where similar methods of drying were used [5,12,15]. The mathematical structure of the first model indicates that the decrease in the moisture ratio occurs in two phases, which are characterized by the way the drying rate changes with the moisture ratio.

The faster decrease in the drying rate observed in the first phase of the process was associated with the drying parameter *k*_1_ 10 times greater than the value of *k*_2_ (Table 1). The values *b*_1_ and *b*_2_ are ranges of MR values, where they decrease with different drying rates. For comparison: in about 98% of the MR range of convective drying of dehydrated apples in sucrose (SCD), a decrease in MR occurred at a high drying rate, while in the case of drying apples without pre-treatment (RCD) using the same drying method, a decrease in MR value was observed at low drying speed (in about 71% of the MR range).

### 2.2. Dry Matter Content and Water Activity

Table 2 presents the values of dry matter content and water activity in dried apples. All variants were characterized by a substantial amount of dry matter, as well as low values of water activity, which determines longer shelf life and microbiological safety of the product. Dried apples, which were previously osmo-dehydrated in polyols solutions, showed a significantly higher percentage of dry matter content (Table 2). The method of drying had significant influence on dry matter content only in the case of dried apples previously dehydrated in sucrose and erythritol (*p*-Value: 0.000 and 0.032, respectively). Xylitol, used as an osmotic substance during osmotic dehydration, had a significant influence on lowering the value of water activity in the case of dried apple samples obtained by the combined method. Significant influence of the osmotic agent on water activity was also observed in the samples after sonication. The lowest values were noticed for xylitol among CD-samples as well as apple samples that were osmo-dehydrated in sucrose solution for CD/VM samples.

Additional ultrasound application significantly reduced the dry matter content (*p*-Value: 0.000), which resulted in significantly higher values of water activity (*p*-Value: 0.000). This statement was also confirmed by t-Test between non-US vs. US data. The test has been constructed to determine whether the difference between the two means of dry matter content and/or water activity values equals 0.0, versus the alternative hypotheses that the mean dry matter content values of non-US samples are greater than US samples, as well as mean water activity values of non-US samples are lower, compared to US-samples. Since the computed *p*-values were less than 0.05 (*p-*Value: 0.042, t: 1.823; *p*-Value: 0.036, t: −1.908, respectively), we rejected the null hypotheses in favor of the alternative. This is probably due to the fact that during sonication, cavitation bubbles may form in the water, which may collapse, causing cell damage. This may promote leaching and ultimately diffusion of water and dry matter from the tissue into the environment [16]. This observation is in agreement with research carried out by Mierzwa and Kowalski [9], who also observed higher values of water activity in convective dried samples after OD with sonication. Pearson’s correlation, carried out between the obtained values presented in Table 2, showed a strong negative correlation with a correlation coefficient value of −0.74 (*p*-Value: 0.000). This means that the higher the percentage of dry matter, the lower the water activity value in the sample.

### 2.3. Hygroscopic Properties

**Figure 4:**
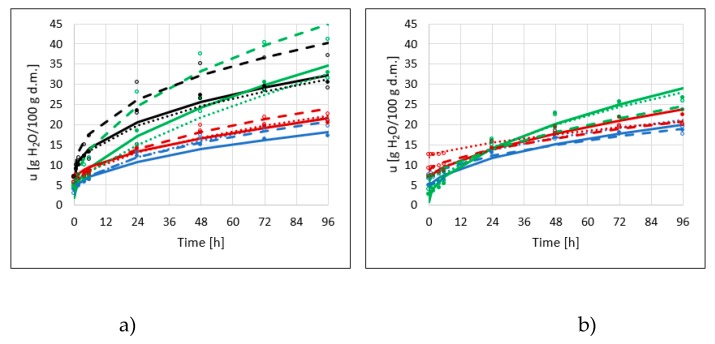
Water adsorption kinetics of dried apples obtained by different drying methods (CD—solid lines, VM—dashed line, CD/VM— dotted line), previously osmo-dehydrated in erythritol (blue lines), xylitol (green lines), sucrose (red lines), and without OD (black lines), without application of ultrasound (**a**) or with US pre-treatment (**b**).

The hygroscopic properties of dried products are associated with the ability to adsorb water in a humid environment [17] and are due to their composition of sugar and pectin in combination with their porous structure. Konopacka et al. [18] reported that at a_w_ below 0.12 apple chips demonstrated excellent crispness. Changes in water content in dried apples were described by equations in the form u = a + bt^c^. After 96 h of adsorption of water vapor from the NaCl solution (aw—0.75), the material reached a significantly different water content. Hygroscopic properties were closely related to the structure of the dried material. The greatest ability to adsorb moisture from the environment was demonstrated by dried apples obtained by the microwave-vacuum method, not subjected to OD (RVM), and dehydrated previously in xylitol solution (XVM)—after 96 h, these samples adsorbed about 40 g of water per 100 g of dry matter (Figure 4a). Water vapor sorption was the most intense in the case of microwave-vacuum drying, which has been proven by other researchers [19]. Slightly less water (about 30 g/100 g d.m.) adsorbed the same type of samples, obtained by convective and combined methods (RCD, RCD/VM and XCD, XCD/VM). These observations also coincide with research carried out by Nowacka and Witrowa-Rajchert [19]. Dried apples, which were osmo-dehydrated in sucrose and erythritol solutions, had about twice the amount of lower hygroscopicity, regardless of the drying method used. The statistical analysis confirmed the significance of all variable factors on the obtained water adsorption values (*p*-Value < 0.05).

Additional application of ultrasounds during the dehydration process significantly affected the changes in the hygroscopic properties of dried apple (Figure 4b). The obtained water adsorption values are much lower in all cases, except for samples containing erythritol (in this case the values were at a similar level). These values may indicate that, during sonication, some damage in plant tissue occurred, resulting in reduced binding capacity of water. Water vapor sorption after the pre-treatment was most intense in the case of apples previously dehydrated in xylitol solution, obtained by convective and combined methods. Fijałkowska et al. [10] also reported that apple tissue, after application of ultrasound, demonstrated lower hygroscopic properties compared to untreated material. However, obtained values of water adsorbed after 72 h were bigger (36–38 g H_2_O/100 g dry matter) than in the present research (Figure 4b). Lower hygroscopicity of the samples indicates greater storage stability for these dried apples.

**Figure 5:**
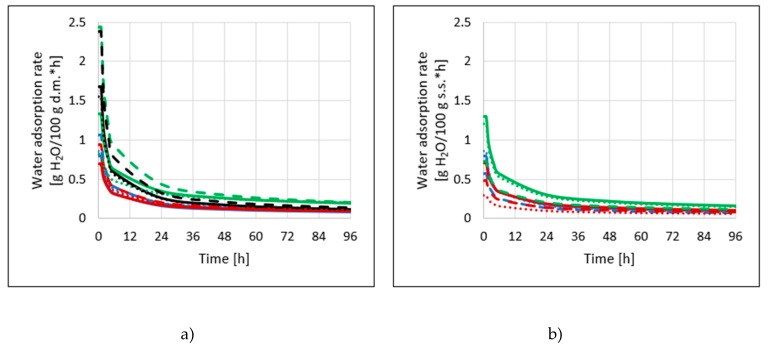
Water adsorption rate of dried apples obtained by different drying methods (CD—solid lines, VM—dashed line, CD/VM—dotted line), previously osmo-dehydrated in erythritol (blue lines), xylitol (green lines), sucrose (red lines), and without OD (black lines), without application of ultrasound (**a**) or with US pre-treatment (**b**).

Differentiation of equations describing changes in water content in dried apples over time has enabled the plotting of water vapor adsorption velocity curves (Figure 5a). Depending on the method of removing water, in the initial period, within one hour, the samples absorbed from 0.7 to 2.4 g water/100 g dry matter. The phenomenon was very intensive during the first 4 h of the process, during which its speed decreased more than twice. Similar values of water vapor adsorption rates for convective dried apples were observed by Witrowa-Rajchert et al. [17]. The rate of water vapor adsorption in apples treated with ultrasound during dehydration was dependent on the drying method. The values were from 0.3 to 1.3 g water/100 g dry matter per hour of the process (in the initial stage) (Figure 5b). The lower water content, as well as the rate of water vapor adsorption in these samples, indicates their weaker hygroscopic properties.

### 2.4. Color Changes

The color is one of the most important parameters, which determines the quality of raw materials and processed products. This characteristic affects the acceptability of a product by the consumer [20]. The lightest color was observed for dried apples without initial osmotic dehydration, subjected to convective (RCD) and combined drying methods (RCD/VM, Table 3). There were no significant differences in surface brightness between the other apple-dried variants. One-way ANOVA analysis of variance indicated a significant impact of the drying method (*p*-Value: 0.004) only in the case of samples without OD, in general, VM samples had a darker color compared to the other two methods. The impact of sonication was also significant (*p*-Value: 0.001), after which the dried samples also had a darker color. It was also confirmed by the t-Test (**p**-Value: 0.003, t: 3.104)—we rejected null hypothesis in favor of the alternative; that means of L* parameter of US-samples are smaller. The same observation was reported by Fijalkowska et al. [20] using the same ultrasound frequency. The L* parameter was influenced by the type of osmotic substance (*p*-Value: 0.027 and 0.000 for Non-US/US samples, respectively) only in the case of CD-samples. Control samples were characterized by the lightest surface of apple tissue.

Color changes after the technological process in relation to the appearance of a fresh apple are presented in Table 3. Definitely, the most similar appearance to raw material was demonstrated in the samples EVM (obtained after dehydration in erythritol and subjected to microwave-vacuum drying), followed by ECD samples, so dehydrated in the same polyol, but dried by convection. The most significant change in the appearance (absolute color differences) was observed in the RVM apples, which also coincides with the brightness observations for this sample. Statistical analysis showed that all factors had a significant impact on obtained ∆E values. The combined drying method turned out to be the most advantageous due to the smallest color changes after the process, while the highest ∆E values were obtained after the microwave-vacuum drying process (*p*-Value: 0.020). Statistical analysis indicated significant influence of the drying method on ∆E values only in the case of control samples (*p*-Value: 0.002). Among the osmotic substances tested, erythritol enabled the most similar appearance of dried apples, compared to the raw material (*p*-Value: 0.006). However, significant influence of osmotic solution was observed only in the case of Non-US samples, which were dried by VM methods and in CD-samples obtained with additional sonication. Application of ultrasounds resulted in significantly greater changes in the color of dried apples (*p*-Value: 0.000). In this case, the t-Test has been constructed to determine whether the difference between the two means equals 0.0 versus the alternative hypothesis—that the difference is less than 0.0. Since the computed P-value was less than 0.05 (*p*-Value: 0.000; t: −4.040), we rejected the null hypothesis. This result corresponds with other research [9]. Moreover, Fijalkowska et al. [20] indicated that lower frequency of ultrasound caused larger alterations of color.

### 2.5. Selected Chemical Analysis

Based on previous research, apples dried by the combined method (CD/VM) were selected for determining the impact of the kind of chosen osmotic substance on selected chemical properties of dried material. Products obtained after combination of osmotic dehydration, with pre-drying of raw materials by the convective method before VM finish-drying, were characterized by a higher quality, compared to other methods of drying [21]. Additionally, properly applied VM finish-drying may favor formation of biologically active constituents that were not present in the raw material [4], simultaneously providing the crispy texture of the final product [13]. This high crispiness intensity can be produced by the high temperature reached during CD/VM, leading to simultaneous high porosity and low moisture content [21]. Different types of osmotic solution were used in the osmotic dehydration before the drying process. A one-factor analysis of variance made it possible to determine the effect of the applied hypertonic solution before the drying process on the obtained values of chemical indicators contained in Table 4. The same letter next to the result in the same row means no statistically significant differences between the values.

Osmotic dehydration in polyols solutions results in changes in the sugar profile in apple tissue [22]. Comparing dried apples, previously dehydrated with a control-dried sample (RCD/VM), there was a decrease in the percentage of protein, ash, dietary fiber, fructose, and glucose. This can be explained by the increasing amount of osmotic substances, which penetrated from the solution into the apple tissue during dehydration. There were no statistically significant differences in fat content between different dried samples. The sodium content in the dried apple containing erythritol was at a similar level as in the case of the control samples, whereas in the dried apples containing xylitol and sucrose, the values were significantly higher. The energy value of the control sample was determined at a similar level as in the dried apple previously subjected to osmotic dehydration in 50% sucrose solution. The use of sugar alcohols, such as xylitol and erythritol as osmo-active substances, made it possible to obtain dried apples with significantly lower calorific value. The largest reduction in energy value was noted in the case of the erythritol solution.

### 2.6. Product Safety

Although acceptable daily intake (ADI) dose has not been specified for them, it is well established that the consumption of excessive amounts of polyols, and slowly digestible carbohydrates can provoke undesirable intestinal side effects, such as flatulence, abdominal cramps, laxation, and—in extreme cases—watery diarrhea. Some of these symptoms are the results of osmotic effects, and others are the results of the fermentative degradation of these compounds in the colon [23,24]. Therefore, in order to ensure consumers have adequate information, products containing more than 10% added polyols must include the advisory statement “excessive consumption may produce laxative effects” (in accordance with European Commission Directive 94/54/EC) [25]. The study on a group of volunteers (men) confirmed the thesis that repeated ingestion of erythritol at a daily dose of 1 g/kg body weight is well tolerated by the body [23]. It was also found that erythritol is characterized by similar tolerability between children and adults in terms of laxation on body weight basis, and the distribution of this substance in the human body is identical, regardless of the consumer’s age [26]. Additional studies have shown that females are more resistant than males to laxative effects when exceeding the safe limit for erythritol intake; 50% of laxative effects occurred at a dose of 1.58 g/kg body weight per day, whereas for men, it was a dose of 1.07 g/kg body weight (bw)/day. The same study also indicates that the diarrhea effect of sorbitol is stronger compared to erythritol, and that sucrose intake at a dose above 1.2 g/kg/day does not cause such problems [27]. Bornet et al. [28] observed biliousness in the form of nausea, rumbling in the stomach, flatulence, and soft feces after ingestion of erythritol at 0.4 and 0.8 g/kg bw by healthy people; however, the biliousness was not significantly different from those after taking equivalent doses of sucrose. Other studies indicate laxative threshold for erythritol (the maximum dose at which no individual responds with laxation) of 0.78 g/kg bw [29]. This indicates the ambiguity of literature reports regarding the maximum doses of polyol intake. Even fewer publications relate to experiments with xylitol intake. The most common is the safe value of daily xylitol intake by adults, up to 100 g [30,31]. Four out of 13 children had diarrhea after consuming over 65 g of xylitol per day [32]. Research conducted by Oku and Nakamura [33] indicates the following doses of xylitol intake: 0.38 g/kg and 0.42 g/kg, and erythritol: 0.46 g/kg and 0.68 g/kg for men and women, respectively.

On the basis of the above-mentioned research, it is worth estimating the safety of consumption of dried apples (Table 5). The following assumptions were made: an adult woman weighs 60 kg, an adult man 75 kg, and a 10-year-old child 30 kg. It is also assumed that the unit of package will contain 15 g of dried apple (it is lightweight after drying).

Any food ingredient supplied to the body in excessive amounts can be toxic to the body. Based on the above calculation (Table 5), it is assumed that a healthy, rationally thinking person is not able to exceed the safe amount of snack consumption in the form of dried apples, so the product has been recognized as safe for the consumer.

### 2.7. Descriptive Sensory Analysis (DSA)

**Figure 6:**
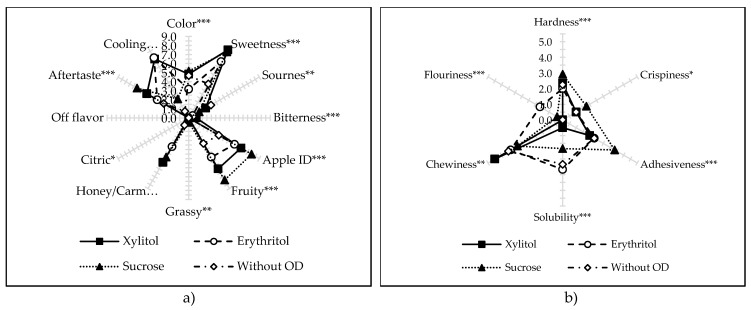
Descriptive sensory analysis of dried apples previously osmo-dehydrated in different solutions (or without OD): attributes for appearance, basic taste, and flavor (**a**), texture attributes (**b**). *, ** and *** -- significant at *p* < 0.05, 0.01, and 0.001, respectively.

Table S1 presents selected qualitative factors of the analyzed dried apple, due to the type of osmotic substance used before drying. Figure 6 only depicts descriptors that were considered statistically significant. There were no statistically significant differences between the values of sensory attributes between samples obtained by different drying methods; therefore, their values were not given. The only exception is the attribute “Off-flavor”, which was not statistically significant; however, it was placed on the chart because it contains valuable information. All variants of dried apples were characterized by a lack of foreign taste, which included the following aromas: hay, wood, earthy, and burnt.

The lowest intensity of the yellow color was demonstrated by samples that had been dehydrated previously in the erythritol solution (Figure 6a)—the samples were characterized by a clearly lighter color, which was also confirmed in the paragraph with color changes (2.4). Apples subjected to the OD before the drying process showed about twice the sweetness, compared to the untreated dried fruit (Table S1). The sour taste was barely discernible and was mainly noted in the samples without OD and using xylitol. This osmotic substance also influenced the occurrence of bitter taste; however, with a slight intensity (value <1). The most intense aroma, reminiscent of fresh apple (apple ID), as well as the fruity flavor was found in the case of dried apples obtained with the use of sucrose. Grass and lemon aromas were virtually undetectable in the samples, with all values being below one. A fairly intense honey/caramel flavor was noticeable in apples dehydrated in sucrose and xylitol solutions. A two-times bigger aftertaste was observed after consuming dried apples that had been previously osmo-dehydrated in sucrose solution, compared to dried raw material. The use of polyols as osmoactive agents in the production of dried apples enabled an interesting property—the cooling effect felt in the mouth while consuming a snack. A very intense effect was noted in both cases of sugar alcohols (erythritol, xylitol). There were no significant differences in the intensity of this effect between these variants. The occurrence of such an effect has also been documented in the literature [24] and is explained by the presence of high negative heat of the solution after dissolution: erythritol −45 kcal / kg, xylitol: −35 kcal / kg [34]. The texture attributes of the descriptive sensory analysis show the relationship between crispiness and hardness of dried apples—the highest values were observed in the samples containing sucrose (Figure 6b). However, values of crispiness were low (Table S1), which are related with water activity around 0.3. The researchers [18] assumed that in order to be accepted by a consumer, it should be scored at least 6 points on the 10-point scale (which means that dried product must have water activity below 0.2). It was also not surprising to obtain the highest adhesiveness values for dried apples containing sucrose. Control samples, and dehydrated in erythritol solution, showed the highest solubility in saliva. Noticeable chewiness was noted in the case of apples previously dehydrated in xylitol, and significant flouriness of samples in dried products containing erythritol.

**Figure 7:**
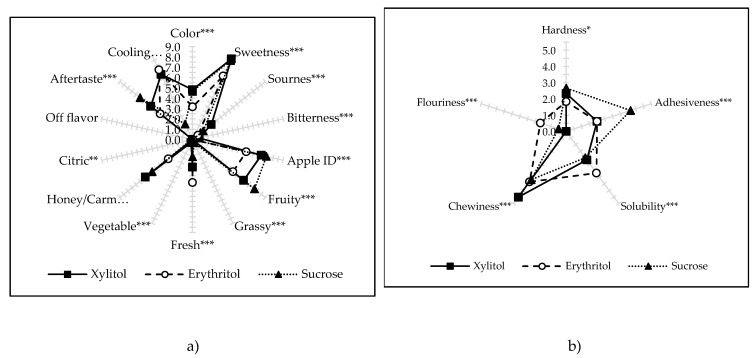
Descriptive sensory analysis of dried apples previously osmo-dehydrated in different solutions with ultrasound pre-treatment: attributes for appearance, basic taste, and flavor (**a**), texture attributes (**b**). *, ** and *** -- significant at *p* < 0.05, 0.01, and 0.001, respectively.

The use of pre-treatment in the form of additional ultrasound application has changed some descriptor values of descriptive sensory analysis (Table S2). Fresh, and vegetable aromas, turned out to be statistically significant, while in the crispiness of dried apples obtained using different osmotic agents, no differences were noted. Similarly, to the dried apples not subjected to sonication, a product containing erythritol showed the lightest color. Basic flavors (sweet, sour, bitter) were similarly assessed as in the case of previous dried samples (without the application of ultrasound). Similarly, the most intense fruit flavor (and apple aroma) was noted in the material obtained using sucrose (Figure 7a). The pre-treatment in the form of ultrasound contributed to the appearance of aromas characterized as “fresh” in dried apples, an effect that was the most intense in the case of samples with erythritol. A slightly palpable vegetable aroma was also observed in the samples containing xylitol. No foreign taste was noted in any dried apples variant, also, when sonication was used. The cooling effect of the products containing polyols was still intense, while slightly less noticeable in dried apples osmo-dehydrated in sucrose solution. The texture attributes of the descriptive sensory analysis were evaluated similarly to the material obtained without sonication. The only difference was in increasing the value of solubility in saliva, in dried apples previously dehydrated in xylitol, subjected to ultrasound (Figure 7b).

**Figure 8:**
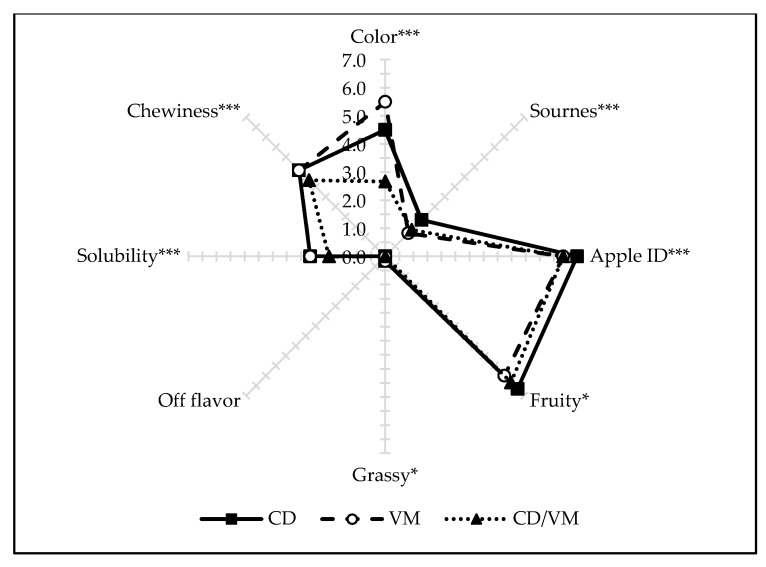
Descriptive sensory analysis of dried apples previously osmo-dehydrated in different solutions with ultrasound pre-treatment for appearance, basic taste, flavor, and texture attributes. *, ** and *** -- significant at *p* < 0.05, 0.01, and 0.001, respectively.

In addition, sonication influenced the diversity of the values of sensory attributes in terms of the drying method used, and mutual interactions between factors, osmotic substance-drying method. For this reason, Table S3 is additionally attached, showing the same sensory attributes due to the drying method used. Still, a small number of descriptors was statistically significant, that is why Figure 8 combines sensory attributes from different groups (appearance, aroma, texture) on one radar chart. The lightest color appeared in dried samples obtained by the combined method (CD/VM), while the most intense were the dried apples obtained by the microwave-vacuum method (VM). The feeling of a sweet and bitter taste did not depend on the drying method used, while the bitter taste was more intensively felt in the case of convective dried apples (CD). At the same time, this material was characterized by the greatest similarity of the aroma to the classic apple flavor (apple ID) and characterized as fruit flavor (Figure 8). It is also worth noting that the occurrence of the cooling effect was only affected by the type of osmotic substance (sugar alcohols) used, whereas the drying method did not significantly change these values. Dried apples obtained by convective and microwave-vacuum methods showed significantly higher solubility and chewiness, compared to dried samples obtained by the combined method (Figure 8).

## 3. Materials and Methods

### 3.1. Sample Preparation

Fresh apples of the Elise variety were collected from the Experimental Fields (Orchards) of the Faculty of Horticulture and Landscape Architecture (Warsaw University of Life Sciences, Warshaw, Poland). The fruits were stored at 4 ± 1 °C and relative humidity of 85–90% in a refrigerator until use. Before the experiment, the apples were washed, stoned, and cut into 5 mm thick slices, and then each slice for 4 pieces.

### 3.2. Pre-Treatment Procedure

Methodics for osmotic dehydration, as well as the optimal process conditions—time and concentration—were selected for testing based on preliminary experiments in a previous work published by Cichowska et al. [7,8]. The 60 g ± 3 g of samples were placed in a 300 mL beaker into syrups in the ratio of 1:4 (fruit:solution) in order to avoid significant changes in the solution concentration. Osmotic solutions were prepared with selected substances from the polyol group (concentration 30%): erythritol and xylitol (Brenntag, Kędzierzyn-Koźle, Poland) as well as sucrose (concentration 50%) dissolved in distilled water. The temperature of the water bath was constant (40 °C). Osmotic dehydration (OD) was conducted for 120 min. Simultaneously, the second experiment was carried out: sonication in osmotic solutions was applied in ultrasonic bath MKD-3 (MKD Ultrasonics, Stary Konik, Poland, internal dimensions: 240 × 140 × 110 mm) for 30 min, at 40 °C and later beakers with samples were transferred to the water bath for additional time of 90 min. During sonication in OD solutions, no significant temperature changes were observed (±1 °C). The frequency used was 21 kHz and the total power generated by sonotrodes was 320 W, which corresponded to the ultrasound intensity of 8 W per gram of material. Afterwards, samples were removed from the osmotic solution, blotted with absorbent paper to remove osmotic liquid from their surface, and were weighed with the accuracy of ±0,01 g. The pre-treatment procedure was conducted one day before drying and stored in the refrigerator at 4 °C. Next day material was transferred quantitatively to the selected drier. After OD, dry matter content of the samples was determined in two repetitions.

### 3.3. Drying Procedure

Apple slices were subjected to drying with three methods. The first method was hot air convective drying (CD), the second one was vacuum-microwave drying (VM), while the third one was a combination of CD/VM. The apple slices obtained without pre-treatment (OD) and dried with different methods were considered to be control samples. Convective drying was performed in a drier designed and built in the Institute of Agriculture Engineering (Wrocław, Poland). The air temperature and velocity were 70 °C and 0.6 m/s, respectively. The portions of apple samples were spread on a round 100 mm tray in a single layer (the tray was loaded with about 2.55 kg/m^2^). The tray was placed on the top of a drying pipe. The convective drier, equipped with six pipes, enabled simultaneous drying of six portions. During the drying, the loss of material’s mass was noted using analytical scale (Axis A5000, Radwag, Radom, Poland) as follow: every 5 min during the first half hour, then every 10 min up to two hours, every 15 min up to 4 h, and then every half an hour. The process was stopped after achieving constant weight. The second type of apple samples were dried in the vacuum-microwave drier SM-200 (Plazmatronika, Wrocław, Poland; schematic diagram [5]). The samples were placed in a container made of organic glass with volume of 0.0068 m^3^. This container was connected to a vacuum system, which consisted of vacuum pump (“BL 30P, Tepro”, Koszalin, Poland), vacuum gauge (“MP 211, Elvac”, Bobolice, Poland), and compensation reservoir with volume of 0.15 m^3^. The pressure in the container varied from 4 to 6 kPa. The maximum temperature of apple samples was controlled and measured using infrared camera i50 (Flir Systems AB, Stockholm, Sweden), directly after taking out samples from the drier (after 4 min cycles). Simultaneously, the weight of the samples was measured. Two levels of microwave powers were used: 240 W during the first 4 cycles (to attain of the critical temperature of sample in the most warm place: 70 °C); afterward, the power was reduced to 60 W and was used in the next cycles until complete drying of biological material. Together with reducing the power of the microwaves, the rotation of a container was applied, providing mixing of samples and necessary to avoid local over-heating of apple slices while drying. Combined drying CD/VM consisted with convective drying for 2 h and pre-dried samples were additionally dried by VM at 60 W. Each type of drying was conducted in two technological repetitions.

### 3.4. Mathematical Modeling

Table Curve 2D version 5.01 (SYSTAT Software Inc., Chicago, IL, USA) enabled prediction of moisture ratio (MR) by fitting the mathematical model to experimental points with the possible highest values of the determination coefficient R^2^ and the lowest values of RMSE. MR was expressed as the ratio of the actual moisture content to the initial moisture content (Equation (3)):

(3) $$MR=\frac{M}{M_{0}}$$

where M is the actual moisture content and M_0_ is the initial moisture content [35].

The value of M_0_ amounting to about 6.62 g/g d. m was determined gravimetrically, taking into account the mass and dry matter content in a sample after drying in a convective drier (SUP-65 WG WAMED, Warsaw, Poland) at the temperature of 70 °C for 24 h. The values of M were predicted based on M_0_ and the actual mass of the dried samples. The final moisture content M_f_ was verified gravimetrically. The mass of samples was measured using an analytical balance XA 60/220X (Radwag, Radom, Poland) with an accuracy of 0.001 g.

### 3.5. Water Activity 

Water activity was determined in dried samples using the device AquaLab CX-2 (Decagon Devices Inc., USA) apparatus, in accordance with the manufacturer’s instruction. The temperature of water activity determination was constant (25 °C). Each measurement was conducted in 4 repetitions.

### 3.6. Hygroscopicity

Hygroscopic properties, expressed as water vapor adsorption kinetics were determined by weighing the dried material with accuracy up to ± 0.0001 g on analytical scale type AE 204S (METTLER Company, Columbus, Ohio, USA) and then placed in a desiccator over saturated NaCl solution in an environment with a_w_ = 0.75. Kinetics were determined for 72 h at a constant temperature of 25 °C. The samples were re-weighed after 0, 1, 2, 4, 6, 24, 48, 72, and 96 h. The measurement was performed in two repetitions for each kind of apple samples.

### 3.7. Color Measurement

Color analysis of the surface of the dried samples was determined with the use of Minolta Chroma Meter CR-200 (Minolta Corp., Osaka, Japan). The measurement conditions were: D65 standard illuminate, 2° Standard Observer, measurement diameter: 30 mm. The results were presented using the directly measured parameters: L* (lightness/darkness), a* (red/green), b* (yellow/blue). The measurements were made in 6 repetitions for every sample; the mean values were reported. The total color differences (ΔE – Equation (4)) were calculated according to the following formula:

(4) $$\Delta E=\sqrt{{\left(\Delta L^{*}\right)}^{2}+{\left(\Delta a^{*}\right)}^{2}+{\left(\Delta b^{*}\right)}^{2}}$$

where ΔL*, Δa*, Δb* – the change of L*, a*, and b* parameter between raw material and samples after drying.

### 3.8. Descriptive Sensory Analysis (DSA)

Seven trained panelists (five women and two men) from the Department of Agro-Food Technology (Miguel Hernández University, Orihuela, Spain) participated in this study. The panel was selected and trained following the ISO standard 8586-1 (1993), and it is specialized in descriptive sensory evaluation of fruits and vegetables. The following attributes were chosen on the basis of the lexicon by [36]: (appearance) color; (basic tastes and chemical feelings) sweetness, sourness, bitterness, and astringency; (flavor) apple ID, fruity, grassy, fresh, vegetable, honey/caramel, citric, off-flavors, hay-like, earthy, woody, burnt, aftertaste, cooling effect; (texture) hardness, crispiness, adhesiveness, solubility in saliva, chewiness, and flouriness. The panel used a numerical scale for quantifying the intensity of the dried apples attributes where 0 represents none and 10 extremely strong with 0.5 increments. The samples were served into odor-free, disposable 90 mL covered plastic cups, at room temperature, and were coded using three-digit numbers. Unsalted crackers and distilled water were provided to panelists to clean their palates between samples.

### 3.9. Selected Chemical Analysis

Total protein content (N × 6.25) was determined by the Kjeldahl method according to PN-A-04018:1975. Total ash content was determined by the weight method according to PN-A-75101/08:1990, pkt. 2 (Test Report N^o^ 21/ZO/2019 dated 09.01.2019) in the Laboratory of Physicochemical and Sensorial Quality of Department of Fruit and Vegetable Product Technology, Warsaw, Poland. Total fat content was determined by titration-extraction method (Soxhlet) according to modification of PN-A-79011-4:1998. Total dietary fiber content was determined by the weight method (after enzymatic hydrolysis), according to AOAC 991.43, AACC 33-07, using Fibertec E Apparatus by FOSS. Total carbohydrate content was calculated from the difference based on the results of moisture, total protein, total ash, total polyols, and total dietary fiber content. Sugar content (fructose, glucose, and sucrose) and polyols content (xylitol, sorbitol, and erythritol) was determined by the HPLC-RID (High Performance Liquid Chromatography – Refractive Index Detectors) method, according to PN-EN 12630:2002 in the Technological Laboratory of Department of Fruit and Vegetable Product Technology, Warsaw, Poland. Sodium content was determined by flame atomic emission spectrometry (FAES) according to procedure *PB-ZA 01 ed. 5 dated 15.03.2016 (Test Report N^o^ 18/ZA/2019 dated 17.01.2019) in the Department of Food Analysis, Prof. Wacław Dąbrowski Institute of Agricultural and Food Biotechnology, Warsaw, Poland. Energy value was calculated based on amount of energy contained in: protein, fat, carbohydrates, polyols, and fiber, according to Regulation (EU) N^o^ 1169/2011 of the European Parliament and of The Council of 25 October 2011. The results represent the means of two independent determinations.

### 3.10. Statistical Analysis

The statistical software Statgraphics Plus ver. 5.1 (StatPoint) and Excel 2016 (Microsoft) were used for data analysis. The influence of pre-treatment (type of osmotic solution, application of ultrasound) as well as method of drying on dependent variables: (WC, a_w,_ hygroscopic properties, color changes) was evaluated by means of a multifactorial analysis of variance (ANOVA) at a significance level α = 0.05. In the case of significant associations, a post-hoc Tukey’s test was performed. In the cases of samples of dried apples, which were selected for chemical and sensory analysis, one-factor (samples) analysis of variance was performed using Statgraphics Plus 5.0 software, whereas post-hoc mean separation was conducted using a Tukey’s multiple range test. Pearson’s correlation coefficient between water activity and moisture content was calculated. The t-Test between non-US vs. US values was also performed.

## 4. Conclusions

The microwave-vacuum drying method allowed for the shortest drying time of dehydrated apples, amounting to 76 min; moreover, the additional application of ultrasounds reduced this time to 36 min. The most popular convective drying was long-lasting; indeed, additional sonication during osmotic dehydration affected the extension of the process time. The combined drying method allowed the total duration of the process to be reduced several times. The use of sugar alcohols (xylitol, erythritol) in the production of dried apples did not badly affect the taste of the obtained dried products. The foreign taste (off-flavor), including the aromas of hay, wood, earthy, and burnt, was imperceptible in all dried samples. The use of polyols as osmoactive agents in the osmotic dehydration process as pre-treatment enabled to obtain a noticeable cooling/refreshing effect felt in the mouth while consuming a snack. The drying method did not have a significant impact on the perception of the intensity of this effect. Additional sonication during osmotic dehydration did not significantly affect attributes of the descriptive sensory analysis for the obtained dried apples. The use of sugar alcohols (xylitol and erythritol) enabled the production of dried snacks with lower calorific value, compared to dried apples obtained by the same method without initial osmotic dehydration. Polyol residues in the product were at a level that guaranteed consumer safety, understood as not exceeding the dose resulting in gastric problems. The best hygroscopic properties, ensuring the storage stability of the dried product, due to the low ability to absorb water from the environment, was observed in dried apples that were previously osmo-dehydrated in erythritol and sucrose solutions.

## Figures and Tables

**Table 1:** Values of the parameters *b*_1_, *k*_1_, *b*_2_, *k*_2_, R^2^, and RMSE of the functions describe the drying kinetics, as well as total drying time of apple samples dried by different methods.

Sample Code	Model	*b_1_*	*k_1_*	*b_2_*	*k_2_*	*R^2^*	RMSE	Drying Time [min]
**Non-US**								
**Erythritol**								
CD	two-term exponential	0.8304	0.0251	0.1658	0.0064	0.9998	0.0037	405
VM	two-term exponential	0.5857	0.1373	0.4381	0.1373	0.9887	0.0345	76
CD/VM	two-term exponential	0.0549	0.1665	0.0263	0.0093	0.9992	0.0090	184
**Xylitol**								
CD	two-term exponential	0.5670	0.0316	0.4486	0.0319	0.9990	0.0093	315
VM	two-term exponential	0.5960	0.1172	0.4321	0.1172	0.9893	0.0342	76
CD/VM	two-term exponential	0.0190	0.0144	0.0190	0.0125	0.8953	0.0018	184
**Sucrose**								
CD	two-term exponential	0.9779	0.0376	0.0256	0.0022	0.9997	0.0048	375
VM	two-term exponential	0.6467	0.1375	0.3707	0.1373	0.9913	0.0308	76
CD/VM	two-term exponential	0.0305	0.1018	0.0498	0.0084	0.9977	0.0005	184
**Without OD**	two-term exponential	0.3843	0.0940	0.6628	0.0939	0.9869	0.0378	84
CD	two-term exponential	0.3411	0.0255	0.7059	0.0257	0.9936	0.0255	345
VM								
CD/VM	Henderson–Pabis	0.0221	0.0211	–	–	0.9113	0.0016	168
**US**								
**Erythritol**								
CD	two-term exponential	0.9262	0.0319	0.0786	0.0011	0.9997	0.0045	615
VM	two-term exponential	1.0097	0.1389	0.0164	0.0001	0.9893	0.0321	36
CD/VM	two-term exponential	−0.2943	0.0399	0.3452	0.0399	0.9443	0.0063	140
**Xylitol**								
CD	two-term exponential	1.0115	0.0319	0.0134	−0.0007	0.9986	0.0103	465
VM	two-term exponential	0.9930	0.1225	0.0510	0.1230	0.9785	0.0447	36
CD/VM	Henderson-Pabis	0.0810	0.0298	-	-	0.9868	0.0025	168
**Sucrose**								
CD	two-term exponential	0.9513	0.0253	0.0390	0.0024	0.9996	0.0054	615
VM	two-term exponential	0.9908	0.1657	0.0256	0.0001	0.9890	0.0277	32
CD/VM	two-term exponential	0.0548	0.0498	0.0394	0.0000	0.9895	0.0011	136

US – osmotic dehydration with ultrasound pre-treatment, Non-US – osmotic dehydration without ultrasound pre-treatment, CD – convective drying, VM – vacuum-microwave drying, CD/VM – combined drying (convective-vacuum-microwave drying).

**Table 2:** Dry matter and water activity in dried apple samples. Means within one column with a different letter superscript are significantly different – homogeneous groups (*p* < 0.05). Lowercase (a,b and c)—factor: osmotic substance/without OD, among the same drying method; uppercase (A,B and C) – factor: drying method, among the same osmotic substance).

	Dry matter [%]		Water Activity [-]	
	Non-US	US	Non-US	US
**Erythritol**				
CD	96.37 ± 0.19 ^c, A^	95.37 ± 0.04 ^b, A^	0.296 ± 0.00 ^a, B^	0.316 ± 0.01 ^b, A^
VM	96.92 ± 0.31 ^d, B^	93.10 ± 1.20 ^a, A^	0.256 ± 0.03 ^a, A^	0.404 ± 0.08 ^a, A^
CD/VM	96.38 ± 0.32 ^c, A, B^	93.95 ± 1.63 ^b, A^	0.284 ± 0.01 ^b, A, B^	0.342 ± 0.03 ^b, A^
**Xylitol**				
CD	95.35 ± 0.18 ^b, A^	97.40 ± 0.12 ^c, A^	0.298 ± 0.01 ^a, A^	0.217 ± 0.04 ^a, A^
VM	96.05 ± 0.26 ^c, A^	93.62 ± 1.70 ^a, A^	0.285 ± 0.00 ^a, A^	0.329 ± 0.01 ^a, B^
CD/VM	96.56 ± 0.52 ^b, c, A^	96.32 ± 1.66 ^b, A^	0.295 ± 0.00 ^a, A^	0.225 ± 0.01 ^a, A^
**Sucrose**				
CD	93.40 ± 0.15 ^a, A^	93.34 ± 0.43 ^a, C^	0.298 ± 0.01 ^a, A^	0.331 ± 0.01 ^b, A^
VM	94.75 ± 0.02 ^b, B^	91.61 ± 0.24 ^a, B^	0.277 ± 0.03 ^a, A^	0.345 ± 0.01 ^a, A^
CD/VM	94.80 ± 0.16 ^b, B^	88.85 ± 0.53 ^a, A^	0.289 ± 0.01 ^b, A^	0.392 ± 0.01 ^c, B^
**Without OD**				
CD	93.74 ± 0.31 ^a, A^	–	0.287 ± 0.01 ^a, A^	–
VM	93.69 ± 0.41 ^a, A^	–	0.288 ± 0.00 ^a, A^	–
CD/VM	93.91 ± 0.47 ^a, A^	–	0.284 ± 0.00 ^a, b, A^	–

US – osmotic dehydration with ultrasound pre-treatment, Non-US – osmotic dehydration without ultrasound pre-treatment, CD – convective drying, VM – vacuum-microwave drying, CD/VM – combined drying (convective-vacuum-microwave drying).

**Table 3:** Values of the L * parameter characterizing brightness and absolute color differences (∆E) in dried apple obtained by various methods. Means within one column with a different letter superscript are significantly different – homogeneous groups (*p* < 0.05). Lowercase (a, b and c) —factor: osmotic substance/without OD, among the same drying method; uppercase (A and B) – factor: drying method, among the same osmotic substance).

	L*		∆E	
	Non-US	US	Non-US	US
**Erythritol**				
CD	80.32 ± 1.57 ^a, A^	74.58 ± 2.79 ^b, A^	4.79 ± 1.34 ^a, A^	14.94 ± 0.59 ^a, A^
VM	82.03 ± 2.29 ^a, A^	72.15 ± 3.81 ^a, A^	2.48 ± 0.33 ^a, A^	15.24 ± 2.14 ^a, A^
CD/VM	76.93 ± 3.01 ^a, A^	75.29 ± 2.72 ^a, A^	7.62 ± 1.81 ^a, A^	11.07 ± 3.34 ^a, A^
**Xylitol**				
CD	79.88 ± 0.95 ^a, A^	74.81 ± 1.96 ^c, A^	8.01 ± 0.72 ^a, A^	18.89 ± 1.04 ^c, A^
VM	74.14 ± 0.32 ^a, A^	68.62 ± 6.66 ^a, A^	12.98 ± 1.81 ^a, b, A^	20.09 ± 4.20 ^a, A^
CD/VM	77.27 ± 2.70 ^a, A^	72.72 ± 3.58 ^a, A^	7.66 ± 2.60 ^a, A^	15.28 ± 5.97 ^a, A^
**Sucrose**				
CD	80.49 ± 0.99 ^a, A^	72.55 ± 2.05 ^a, A^	9.72 ± 1.71 ^a, A^	18.45 ± 2.01 ^b, A^
VM	73.42 ± 6.05 ^a, A^	70.32 ± 1.73 ^a, A^	10.07 ± 5.34 ^a, b, A^	17.28 ± 1.43 ^a, A^
CD/VM	76.49 ± 3.83 ^a, A^	75.64 ± 2.23 ^a, A^	9.03 ± 2.02 ^a, A^	12.40 ± 6.04 ^a, A^
**Without OD**				
CD	84.80 ± 0.31 ^b, B^	–	7.87 ± 1.18 ^a, A^	–
VM	68.90 ± 2.77 ^a, A^	–	20.16 ± 1.31 ^b, B^	–
CD/VM	83.79 ± 0.91 ^a, B^	–	7.71 ± 0.60 ^a, A^	–

US – osmotic dehydration with ultrasound pre-treatment, Non-US – osmotic dehydration without ultrasound pre-treatment, CD – convective drying, VM – vacuum-microwave drying, CD/VM – combined drying (convective-vacuum-microwave drying).

**Table 4:** Results of chemical analysis of raw apple tissue, and dried by combined method – previously osmo-dehydrated in erythritol (ECD/VM), xylitol (XCD/VM), sucrose (SCD/VM), without OD (RCD/VM).

	ECD/VM	XCD/VM	SCD/VM	RCD/VM	Raw Apple
**Total protein content (N × 6.25)** [g/100 g]	1.43 ± 0.09 ^a^	1.22 ± 0.03 ^a^	1.52 ± 0.04 ^a, b^	1.77 ± 0.11 ^b^	0.34 ± 0.00
**Total ash** [g/100 g]	0.51 ± 0.00 ^b^	0.47 ± 0.01 ^a^	0.46 ± 0.00 ^a^	0.56 ± 0.01 ^c^	0.17 ± 0.00
**Total fat** [g/100 g]	0.33 ± 0.06 ^a^	0.36 ± 0.09 ^a^	0.25 ± 0.03 ^a^	0.33 ± 0.06 ^a^	0.06 ± 0.00
**Total dietary fiber** [g/100 g]	6.81 ± 0.30 ^a^	7.42 ± 0.08 ^a^	7.23 ± 0.01 ^a^	8.52 ± 0.08 ^b^	1.74 ± 0.03
**Total carbohydrates** [g/100 g]	49.54	49.47	80.84	77.64	14.06
**Sugars:** [g/100 g]	46.55	48.65	78.28	77.56	12.93
**fructose** g/100 g]	22.32 ± 0.11 ^b^	22.46 ± 0.08 ^b^	20.38 ± 0.09 ^a^	36.04 ± 0.25 ^c^	6.86 ± 0.05
**glucose** [g/100 g]	7.39 ± 0.11 ^b^	6.22 ± 0.04 ^a^	6.20 ± 0.07 ^a^	11.06 ± 0.11 ^c^	2.32 ± 0.01
**sucrose** [g/100 g]	16.84 ± 0.05 ^a^	19.98 ± 0.09 ^b^	51.70 ± 0.14 ^d^	30.46 ± 0.04 ^c^	3.75 ± 0.04
**Polyols:** [g/100 g]	34.85 ± 0.29 ^c^	35.79 ± 0.30 ^c^	2.05 ± 0.02 ^a^	3.33 ± 0.08 ^b^	0.68 ± 0.02
**Sorbitol + xylitol** [g/100 g]	2.26 ± 0.04 ^a^	35.79 ± 0.30 ^c^	2.05 ± 0.02 ^a^	3.33 ± 0.08 ^b^	0.68 ± 0.02
**erythritol** [g/100 g]	32.59 ± 0.25 ^b^	0.00 ± 0.00 ^a^	0.00 ± 0.00 ^a^	0.00 ± 0.00 ^a^	0.00 ± 0.00
**Sodium (Na)** [mg/kg]	16.9 ± 0.14 ^a^	19.65 ± 0.21 ^b^	19.95 ± 0.35 ^b^	17.15 ± 0.49 ^a^	2.8 ± 0.14
**Energy value** [kJ/100 g]	225.8	306.7	351.1	345.0	63.3
**Energy value** [kcal/100 g]	955.5	1292.3	1487.8	1460.8	267.7

Means within one row with a different lowercase letters superscript (a, b and c) are significantly different – homogeneous groups (p < 0.05).

**Table 5:** Summary of product safety calculations containing the following polyols.

	Erythritol		Xylitol	
	According [23]	According [33]	According [30,32]	According [33]
**Adult female 60 kg**	60 g of erythritol contains in 184 g of the product → 12 packages	41 g of erythritol contains in 126 g of the product → 8 packages	100 g of xylitol contains in 279 g of the product → 18 packages	25 g of xylitol contains in 70 g of the product → 5 packages
**Adult male 75 kg**	75 g of erythritol contains in 215 g of the product → 14 packages	35 g of erythritol contains in 107 g of the product → 7 packages	100 g of xylitol contains in 279 g of the product → 18 packages	29 g of xylitol contains in 81 g of the product → 5 packages
**10-years girl 30 kg**	30 g of erythritol contains in 92 g of the product → 6 packages	20 g of erythritol contains in 61 g of the product → 4 packages	65 g of xylitol contains in 182 g of the product → 12 packages	13 g of xylitol contains in 36 g of the product → 2 packages
**10-years boy 30 kg**	30 g of erythritol contains in 92 g of the product → 6 packages	14 g of erythritol contains in 43 g of the product → 3 packages	65 g of xylitol contains in 182 g of the product → 12 packages	11 g of xylitol contains in 31 g of the product → 2 packages

## Supplementary Materials

Table S1: Attributes of descriptive sensory analysis of dried apples dehydrated (or not) without the application of ultrasound depending on the osmotic substance used, Table S2: Attributes of descriptive sensory analysis of dried apples dehydrated with the application of ultrasound depending on the osmotic substance used, Table S3: Attributes of descriptive sensory analysis of dried apples dehydrated with the application of ultrasound depending on the drying method used.

## Abbreviations

OD	osmotic dehydration
US	ultrasound
CD	convective drying
VM	vacuum-microwave drying
CD/VM	combined drying (convective-vacuum-microwave drying)

## Appendix A

**Table A1:** Nomenclature.

N^o^	Sample Code	Drying Method	Type of Hypertonic Solution	Sonication
1.	XCD	CD	Xylitol	−
2.	XVM	VM	Xylitol	
3.	XCD/VM	CD/VM	Xylitol	
4.	ECD	CD	Erythritol	−
5.	EVM	VM	Erythritol	
6.	ECD/VM	CD/VM	Erythritol	
7.	SCD	CD	Sucrose	−
8.	SVM	VM	Sucrose	
9.	SCD/VM	CD/VM	Sucrose	
10.	RCD	CD	Without OD	−
11.	RVM	VM	Without OD	
12.	RCD/VM	CD/VM	Without OD	
13.	XCD_US	CD	Xylitol	+
14.	XVM_US	VM	Xylitol	
15.	XCD/VM_US	CD/VM	Xylitol	
16.	ECD_US	CD	Erythritol	+
17.	EVM_US	VM	Erythritol	
18.	ECD/VM_US	CD/VM	Erythritol	
19.	SCD_US	CD	Sucrose	+
20.	SVM_US	VM	Sucrose	
21.	SCD/VM_US	CD/VM	Sucrose	
