# Supplementary material for: Physicochemical Properties of Dried Apple Slices: Impact of Osmo-Dehydration, Sonication, and Drying Methods
Source: Molecules. 2020 Feb 28;25(5):1078. doi: 10.3390/molecules25051078 (PMC7179141; doi:10.3390/molecules25051078)
Supplement: Supplementary file 1 [file molecules-25-01078-s001.pdf]

Table S1. Attributes of descriptive sensory analysis of dried apples dehydrated (or not) without the application of ultrasound depending on the osmotic substance used.

Attribute	ANOVA†	Osmotic agent			
		Xylitol	Erythritol	Sucrose	Without OD
		**Appearance**			
**Color**	***	4.8 a	3.2 b	5.2 a	4.7 a
		**Basic taste**			
**Sweetness**	***	8.7 a	7.2 b	8.4 a	4.3 c
**Sourness**	**	2.2 ab	0.5 c	1.3 bc	2.8 a
**Bitterness**	***	0.8 a	0.0 b	0.0 b	0.0 b
		**Flavor**			
**Apple ID**	***	6.7 b	5.8 c	8.0 a	3.8 d
**Fruity**	***	6.5 b	5.0 c	7.9 a	3.3 d
**Grassy**	**	0.0 ab	0.0 c	0.5 a	0.0 bc
**Fresh**	NS	3.0 a	4.7 a	1.8 a	2.2 a
**Vegetable**	NS	0.5 a	0.0 a	0.3 a	0.1 a
**Honey/Carmel**	***	5.7 a	3.7 b	5.0 a	0.9 c
**Citric**	*	0.0 ab	0.0 b	0.2 a	0.0 a
**Off flavor**	NS	0.0 a	0.0 a	0.0 a	0.0 a
**Aftertaste**	***	5.3 b	4.0 b	6.6 a	3.2 c
**Cooling effect**	***	7.5 a	7.7 a	2.4 b	0.8 c
		**Texture**			
**Hardness**	***	2.3 bc	2.0 c	2.9 a	2.3 b
**Crispiness**	*	1.0 b	1.0 b	1.8 a	1.0 b
**Adhesiveness**	***	2.0 b	2.3 b	3.8 a	2.3 b
**Solubility**	***	0.5 b	3.2 a	1.8 b	2.8 a
**Chewiness**	**	5.0 a	3.8 ab	3.3 c	4.0 bc
**Flouriness**	***	0.0 bc	1.7 a	0.4 b	0.0 c

† NS – not significant at $p = 0.05$; *, ** and *** -- significant at $p < 0.05$, 0.01 and 0.001, respectively; the letters denote homogeneous groups; values followed by the same letter, within the same row were not significantly different ($p = 0.05$), according to Tukey's least significant difference test.

Table S2. Attributes of descriptive sensory analysis of dried apples dehydrated with the application of ultrasound depending on the osmotic substance used.

Attribute	ANOVA†	Osmotic agent		
		Xylitol	Erythritol	Sucrose
**Appearance**				
**Color**	***	4.8 a	3.2 b	4.7 a
**Basic taste**				
**Sweetness**	***	8.7 a	6.8 b	8.5 a
**Sourness**	***	2.3 a	0.7 c	1.3 b
**Bitterness**	***	0.7 a	0.0 b	0.0 b
**Flavor**				
**Apple ID**	***	6.8 b	5.3 c	7.3 a
**Fruity**	***	6.3 b	5.0 c	7.7 a
**Grassy**	***	0.0 b	0.0 b	0.3 a
**Fresh**	***	2.7 b	4.2 a	1.7 c
**Vegetable**	***	0.3 a	0.0 b	0.0 b
**Honey/Carmel**	***	5.8 a	3.0 c	5.0 b
**Citric**	**	0.0 b	0.0 b	0.2 a
**Off flavor**	NS	0.0 a	0.0 a	0.0 a
**Aftertaste**	***	5.2 b	4.0 c	6.5 a
**Cooling effect**	***	7.0 a	7.5 a	1.7 b
**Texture**				
**Hardness**	*	2.3 ab	1.8 b	2.7 a
**Crispiness**	NS	1.0 a	1.0 a	1.3 a
**Adhesiveness**	***	2.0 b	2.0 b	4.2 a
**Solubility**	***	2.2 b	3.2 a	2.0 b
**Chewiness**	***	5.0 a	3.8 b	3.7 b
**Flouriness**	***	0.0 b	1.7 a	0.5 b

† NS – not significant at $p = 0.05$; *, ** and *** -- significant at $p < 0.05$, 0.01 and 0.001, respectively; the letters denote homogeneous groups; values followed by the same letter, within the same row were not significantly different ($p = 0.05$), according to Tukey's least significant difference test.

Table S3. Attributes of descriptive sensory analysis of dried apples dehydrated with the application of ultrasound depending on the drying method used.

Attribute	ANOVA†	Drying method		
		CD	VM	CD/VM
**Appearance**				
**Color**	***	4.5 b	5.5 a	2.7 c
**Basic taste**				
**Sweetness**	NS	8.0 a	8.2 a	7.8 a
**Sourness**	***	1.8 a	1.2 b	1.3 b
**Bitterness**	NS	0.2 a	0.2 a	0.3 a
**Flavor**				
**Apple ID**	***	6.8 a	6.3 b	6.3 b
**Fruity**	*	6.7 a	6.0 b	6.3 b
**Grassy**	*	0.2 a	0.2 ab	0.0 b
**Fresh**	NS	3.2 a	2.7 a	2.7 a
**Vegetable**	NS	0.0 a	0.2 a	0.2 a
**Honey/Carmel**	NS	4.7 a	4.7 a	4.5 a
**Citric**	NS	0.2 a	0.0 a	0.0 a
**Off flavor**	NS	0.0 a	0.0 a	0.0 a
**Aftertaste**	NS	5.3 a	5.2 a	5.2 a
**Cooling effect**	NS	5.3 a	5.8 a	5.0 a
**Texture**				
**Hardness**	NS	2.0 a	2.3 a	2.5 a
**Crispiness**	NS	1.0 a	1.3 a	1.0 a
**Adhesiveness**	NS	3.0 a	2.7 a	2.5 a
**Solubility**	***	2.7 a	2.7 a	2.0 b
**Chewiness**	*	4.3 ab	4.3 a	3.8 b
**Flouriness**	NS	0.7 a	0.8 a	0.7 a

† NS – not significant at $p = 0.05$; *, ** and *** -- significant at $p < 0.05$, 0.01 and 0.001, respectively; the letters denote homogeneous groups; values followed by the same letter, within the same row were not significantly different ($p = 0.05$), according to Tukey's least significant difference test.
